# County-to-county migration modeling in the United States: the effects of data source and model selection

**DOI:** 10.1007/s10109-025-00470-7

**Published:** 2025-07-11

**Authors:** Philip E. Morefield, Timothy F. Leslie

**Affiliations:** 1 Department of Geography and Geoinformation Science, George Mason University, 4400 University Dr., MS 6C3, Fairfax, VA 22030, USA; 2 U.S. EPA, Center for Public Health and Environmental Assessment, Washington, D.C, USA

**Keywords:** Migration, Spatial interaction models, Zero-inflated Poisson, Radiation model, Datasets, R23, C21

## Abstract

Internal migration plays a critical role in shaping demographic and economic landscapes, yet the ability to model migration flows accurately remains a methodological challenge. This study evaluates the performance of different migration models applied to three key U.S. data sources: the Internal Revenue Service (IRS) migration data, the American Community Survey (ACS), and the Census long-form data. While these datasets provide valuable insights into county-to-county migration, they differ in temporal coverage, flow suppression thresholds, and demographic granularity, each introducing unique challenges to migration modeling. Using a comparative framework, this study assesses the impact of data source selection on the accuracy and bias of widely used migration models, including the gravity model, Poisson regression, and the radiation model. Our findings highlight the trade-offs inherent in each dataset, demonstrating that IRS data yield lower prediction errors in aggregate flow estimates but lack demographic specificity, whereas ACS and Census data offer richer demographic detail and capture a larger number of distinct migration streams, though they may introduce noise due to small-flow estimates and suppression thresholds for confidentiality. The results underscore the importance of aligning data selection with research objectives and contribute to broader discussions on best practices for migration modeling.

## 1 Introduction and motivation

Scholars and practitioners have delved into the intricacies of migration, dedicating over a century to investigating, modeling, and predicting this fundamental aspect of human mobility. Migration, broadly defined as the permanent or semipermanent relocation of individuals across administrative boundaries (Morrison et al. 2007), represents a dynamic process shaped by diverse motives and obstacles that has been studied using a variety of theoretical frameworks and empirical models (Christopher and Leslie 2015; Wright and Ellis 2016). This movement, often influenced by socioeconomic, political, and environmental factors, constitutes the most volatile component of population change (Niedomysl 2011; Ahmad Yar and Bircan 2023; Caballero Reina et al 2024).

At the heart of migration, flow modeling lies the gravity model, a seminal framework derived from Newton’s law of gravitational attraction. The roots of this model can be traced back at least 75 years to the pioneering works of Stewart (1941) and Zipf (1946). Zipf’s contribution marked a significant advancement in migration modeling, as his formula for estimating intercity movement of people resonated with Ravenstein’s theory (1889), which posited a connection between migration, population size, and distance. Zipf’s approach laid the groundwork for what is now recognized as the general form of the gravity model equation, a pivotal milestone in the evolution of migration modeling (Poot et al. 2016; Curiel et al. 2018). The gravity model is typically expressed as:

(1) $$M_{ij}=k\frac{P_{i}^{\beta_{1}}P_{j}^{\beta_{2}}}{D^{\alpha }}$$

The basic gravity model.

where $M_{ij}$ denotes the number of migrants relocating from location i to location j, and ${\text{P}}_{i}$ and ${\text{P}}_{j}$ represent the populations at the origin and destination locations, respectively. The variables $\beta_{1}$, $\beta_{2}$, α, and k are parameters to be estimated, encapsulating the intricate dynamics of migration. This model revolutionized migration analysis by enabling the estimation of all its terms through linear regression, thereby facilitating its widespread adoption as a primary tool for estimating migration and other spatial interactions. Despite its age, the gravity model remains a cornerstone in migration research, owing to its intuitive framework and robust analytical capabilities (Cameron and Poot 2024).

In the United States, measuring migration poses a formidable challenge in the scholarly landscape, marked by the absence of a single, comprehensive, authoritative data source (Smith et al. 2013). Nonetheless, researchers frequently rely on three prominent datasets, each offering county-to-county migration information for the entire U.S., albeit with notable disparities. In addition to these flow-based datasets discussed below, public use microdata sample (PUMS) datasets are also commonly used in migration research, particularly for individual-level migration analysis (Mulholland and Hernandez-Julian 2021). However, the PUMS data are aggregated by unique geographic boundaries, complicating both comparisons to other migration flow data sources and the marriage of exogenous variables reported by common administrative boundaries. Recent scholarship has utilized user information from social media platforms like Facebook to measure migration and human mobility (Alexander et al. 2019). However, users of social media are almost certainly not representative of whole populations, necessitating intricate correction techniques, and those data offer little in the way of demographic information beyond age and sex.

The 2000 Census, conducted by the U.S. Census Bureau (2000), stands as a foundational resource, having surveyed 1 out of 6 households at the time to produce and provide detailed data on migration categorized by age and race. However, the decennial census no longer collects such comprehensive migration data, with its responsibilities now assumed by the American Community Survey (ACS; U.S. Census Bureau 2014). Launched in 2005, the ACS represents the most recent and extensive effort to gather migration data, sampling 1% of the U.S. population annually and furnishing rolling five-year averages spanning periods like 2005–2009, 2006–2010, 2007–2011, and so forth. Small migration flows (i.e., less than three individuals from different households) are suppressed to avoid allowing the identification of individuals. While certain iterations of the five-year ACS datasets incorporate demographic attributes such as race, ethnicity, gender, and age, they lack cross-tabulated results (U.S. Census Bureau 2014). An exceptional attribute of the ACS is the inclusion of a 90% confidence interval for each county-to-county migration flow, a feature absent in other data sources. This provision furnishes a measure of uncertainty surrounding migration estimates, thereby enhancing the overall reliability of ACS data.

Another significant data source in migration research is the Internal Revenue Service (IRS; 2016), which has supplied annual migration data since 1983. Derived from changes in household addresses indicated on tax returns, IRS data offers a unique perspective on migration dynamics. Despite its lack of demographic granularity, IRS records include aggregate adjusted gross income for each county-to-county migration flow since 2012, shedding light on the financial implications of migration patterns. Both the IRS and ACS datasets apply suppression rules that censor smaller county-to-county migration flows for privacy reasons, though their methods differ in scope and threshold. The IRS data exclude all migration flows involving fewer than 20 individuals, while the ACS suppresses flows with fewer than three individuals from different households. This distinction means that IRS data systematically omit small migration flows, whereas ACS maintains more granular flows but applies stricter privacy constraints for very low-magnitude moves. These differences have implications for modeling. The IRS suppresses county pairs with fewer than twenty movers, so it omits many low-magnitude flows that cluster at short distances and consequently underestimates nearby migration. ACS data provide richer detail, but the sampling error associated with very small flows can introduce variability in models that are sensitive to flow truncation.

While previous research has raised concerns regarding the utility of ACS (Franklin and Plane 2006; Rogers et al. 2008) and IRS (DeWaard et al. 2022) migration data, there is a dearth of scholarship concerning direct comparisons among these data sources. Understanding migration patterns in the United States necessitates navigating the complexities of these disparate datasets. Despite the significant differences in survey design and data collection methodologies across the IRS, ACS, and Census data sources, the literature is less clear on how different migration models respond to the characteristics and limitations of these datasets, rather than attempting to determine which dataset is most accurate. Even rudimentary descriptive statistics of county-to-county migration datasets from these sources underscore potential quantitative discrepancies. This study examines internal migration at the county-to-county level within the United States, distinguishing it from broader discussions of international or state-to-state migration. Specifically, we aim to answer the following research questions: How do the IRS, ACS, and Census data sources compare in terms of accuracy and bias in migration modeling? What are the implications of data selection for migration model outcomes, particularly in estimating intercounty migration flows? Which migration models best account for dataset-specific idiosyncrasies, such as suppressed small flows or temporal aggregation? Through this analysis, we provide insights into best practices for integrating different data sources into migration research, offering guidance for scholars and policymakers in selecting the most appropriate modeling approaches. This study contributes to migration scholarship by emphasizing the importance of aligning data selection with methodological frameworks.

## 2 Data sources

Despite the longstanding use of ACS, Census, and IRS migration datasets in U.S. internal migration research, the specific trade-offs inherent in these data sources—such as ACS’s rolling multiyear estimates, IRS’s lack of demographic details but greater coverage of total flows, and Census’s decennial resolution—necessitate careful methodological choices (Frey 2011). Researchers must evaluate how these datasets capture different migration processes and whether their respective limitations affect the reliability of statistical models used to estimate flows.

**Fig. 1:**
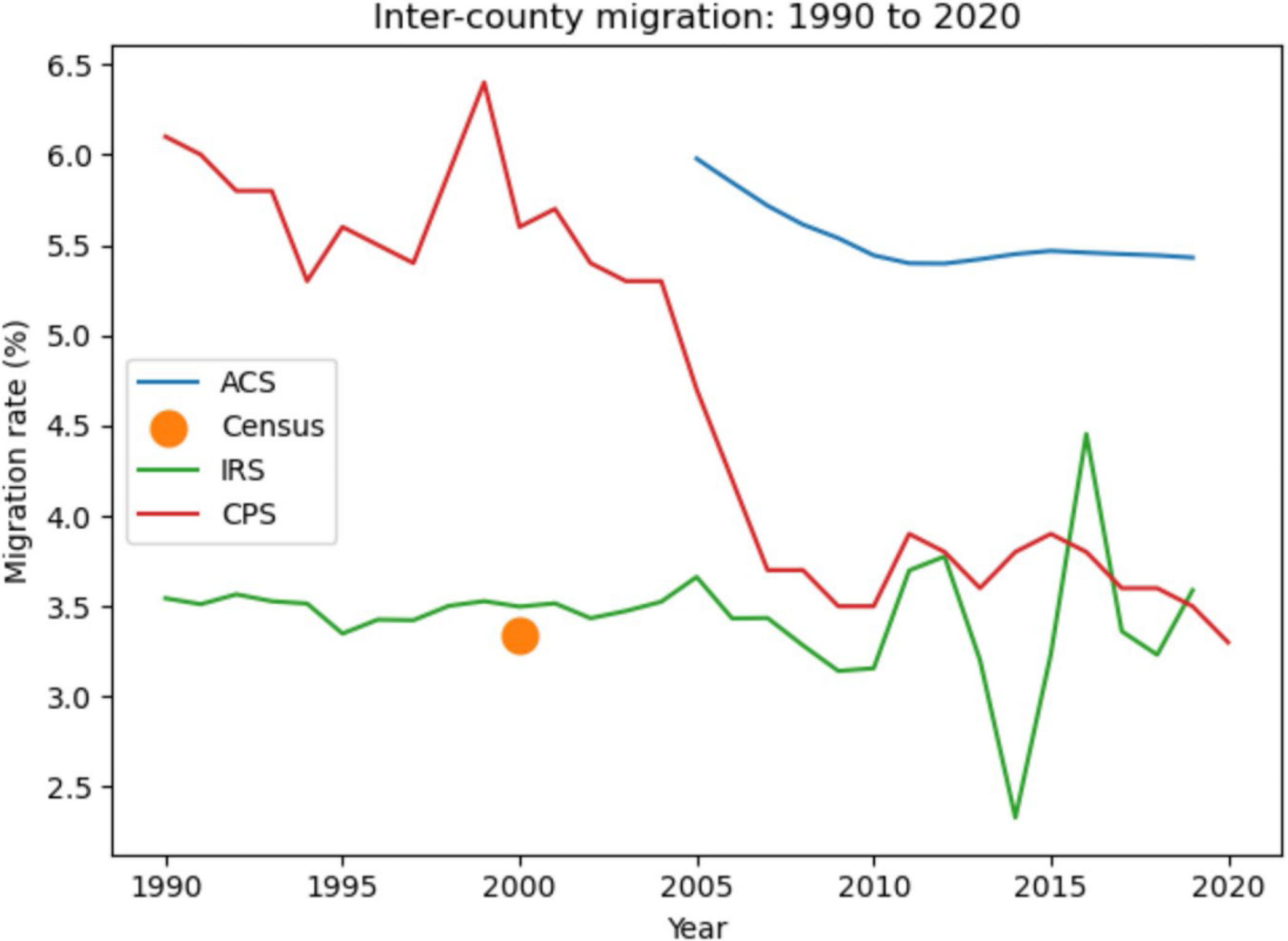
Trends in national migration rate since 1990 by data source. The Current Population Survey (2022) does not include county-to-county migration records and is included in this figure only for reference

Our evaluation of migration models incorporates three distinct sources of migration data to discern variations in results stemming solely from the choice of input data. We limited the scope of our analysis to migration data sources that were free and publicly available without any notable restrictions for use. Table 1 provides an overview of the migration data examined in this study, while Fig. 1 shows the intercounty migration rates from 1990 to 2020 utilizing the breadth of these datasets.

### 2.1 IRS county‑to‑county migration files

The Internal Revenue Service (IRS) provides annual migration counts by correlating the county of residence for income tax filers across consecutive years. Although these counts do not represent individual migration instances but rather the movement of tax exemptions from one county to another, they serve as a valuable proxy for migration patterns. Despite acknowledged limitations, such as the exclusion of residents who do not file tax returns, the annual frequency of IRS data renders it a unique and indispensable resource for migration studies. These data span from 1990 to 2020, albeit without accompanying demographic details of the migrants. To safeguard individual privacy, the IRS suppresses all flows involving fewer than 10 exemptions up to 2012, after which the threshold is increased to 20 exemptions.

### 2.2 American community survey

The American Community Survey (ACS) annually surveys approximately 1% of the U.S. population, offering valuable insights into demographic trends and migration patterns. County-to-county migration data from the ACS are available from 2005 to 2020, with subsequent annual surveys aggregated over rolling five-year intervals. The earliest dataset in this study spans from 2005 to 2009, representing the average annual migration flow between counties during that period. The most recent dataset covers the years 2016 to 2020, resulting in a total of eleven ACS migration files that encompass overlapping time frames.

Notably, the ACS datasets for 2006–2010 and 2011–2015 provide county-to-county migration flows categorized by four race groups (White, Black, Asian, and Other) and Hispanic and Not-Hispanic designations. Hispanic origin is considered an ethnic trait, leading to an overlap between these categories and the four race groups. However, ACS migration flows are not cross-tabulated by race and Hispanic origin, and respondents self-report their race and ethnicity in the survey.

### 2.3. U.S. census bureau enhanced migration files

Before the introduction of the American Community Survey (ACS), demographically detailed migration data were primarily sourced from the enhanced migration files provided by the U.S. Census Bureau. These files were collected as part of the decennial censuses from 1970 to 2000, capturing respondents’ places of residence five years prior to the actual census.

Unlike the annual migration data from the IRS or the average annual flows between counties recorded in the ACS, the Census migration data represent the net migration of individuals over a five-year period (i.e., 1995 to 2000). The 2000 Census migration files report county-to-county migration flows for five race groups: White, Black, American Indian and Alaska Native, and Asian and Pacific Islander. Respondents self-report their race and ethnicity in the survey, and these demographic details are crucial for understanding migration patterns and their sociocultural implications. Like the ACS data, the 2000 Census migration data are not cross-tabulated by race, age, or other demographic characteristics.

### 2.4 Comparison

**Fig. 2:**
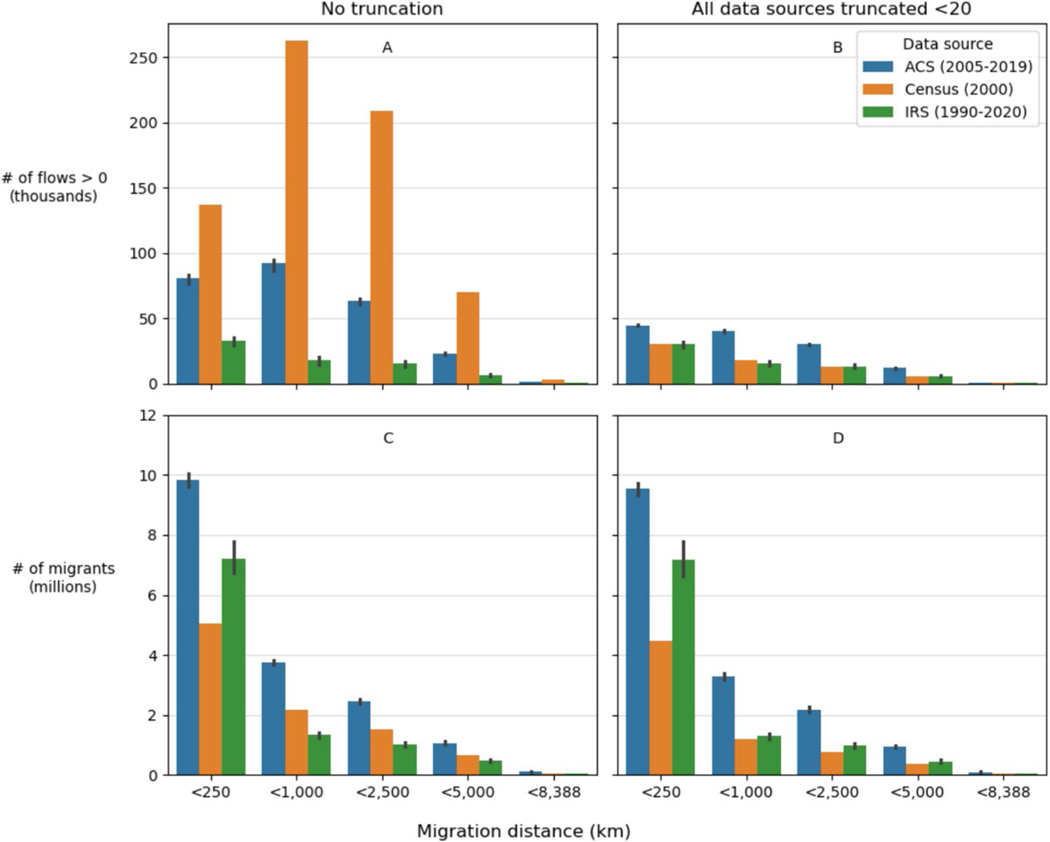
Average annual migration and number of migration flows by distance and data source, with and without truncation

The visualization of migration flow size distribution provides valuable insights into the disparities among the three data sources. Figure 2 illustrates notable differences between the three data sources, particularly with respect to the total number of migration flows. Panel A shows large differences between reported total migration at all distances, with Census flows doubling ACS flows, and four to five times larger than IRS flows, in some cases. Noticeably, these disparities all but disappear when all three data sources are truncated (Fig. 2B), indicating that the Census data include a considerable number of very small migration flows.

This observation is supported by Fig. 2C and D, which demonstrate only small changes in total migration between the full and truncated versions of each data source. These two figures also confirm the similar magnitude of Census and IRS values observed in Fig. 1; however, this relationship does not hold true for migration covering less than 250 km, where IRS data show considerably more migration than Census. The ACS data report more migration than Census and IRS at all distances, which is again consistent with Fig. 1.

## 3 Methodology

Stouffer (1960) introduced the Intervening Opportunities and Competing Migrants model, suggesting that migration is influenced by both geographical distance and the presence of intervening opportunities and competing migrants. Individuals are more likely to move to locations offering greater opportunities and less competition. Empirical studies (Haynes et al. 1973; Miller 1972) supported Stouffer’s model, demonstrating its predictive power and showing it often outperformed the traditional gravity model (Freymeyer & Ritchey 1985; Galle and Taeuber 1966; Wadycki 1975). Consequently, variants of Stouffer’s concepts have become integral to migration and spatial interaction studies (Raphael 1998; Cameron and Poot 2024).

Variations of the Intervening Opportunities term exist, but the general approach involves considering surrounding locations near the origin as potential alternatives. This aligns with Stouffer’s original idea (Stouffer 1940), although he later suggested that only opportunities physically between the origin and destination should be considered (Wadycki 1975). This debate highlights the ongoing refinement of Stouffer’s framework in migration research. The IO approach captures two key concepts: first, the number of intervening opportunities increases with distance, resonating with Ravenstein’s premise that distance is a proxy for the cumulative number of opportunities. Second, migration likelihood decreases as the number of intervening opportunities increases. This aligns with the principle that individuals prefer the shortest possible distance for opportunities, reflecting a natural inclination toward convenience and efficiency in migration decisions.

Building upon the framework established by Raphael (1998), we adopt Stouffer’s intervening opportunities (S), which we define as:

$$S_{ij}=\sum_{k}P_{k}\forall k|d_{ik}<d_{ij}$$

where ${\hat{M}}_{ij}$ is the number of migrants between the origin i and the destination j; $M_{i}$ is the number of migrants originating from location i; and P is the population at a location.

We conducted estimations of the migration models using the three distinct data sources, each encompassing a range of one (Census), 11 (ACS) or 30 (IRS) years of data. Each data source offers distinct identifiers for the origin county (i), destination county (j), the annual number of migrants (M), and the year of migration (t). The estimation process entails employing county population (P) for a given year (t) and the number of annual migrants (M) at time (t+1) between the origin county (i) and destination county (j) for all unique county pairs. This approach is designed to mitigate confounding or mis-specification errors, as it ensures that the population of a county at year (t) is partially influenced by migration for year (t). Subsequently, we validate each model by comparing the estimated number of migrants ${\hat{M}}_{ij}$ for each unique county-to-county pair at year (t+1) to the observed migration $M_{ij}$, t+1.

To comprehensively evaluate their effectiveness, we employ four distinct modeling processes, each serving as a robust platform for assessing the impact of alternative deterrence measures. These measures involve substituting Euclidean distance with population-weighted distance measures, which incorporate demographic density as a spatial adjustment factor (Niedomysl et al 2017). This approach aligns with recent migration modeling literature emphasizing accessibility-adjusted distance metrics (Cameron and Poot 2024; Drouhot et al 2023). Subsequently, we systematically repeat the process of model estimation and validation across all three data sources to ensure the reliability and consistency of our findings. Ordinary least squares (OLS), and to a lesser extent Poisson, models have a well-established track record of applications in peer-reviewed migration research. Their extensive use underscores their relevance and reliability within our analytical framework.

Alongside these conventional models, we also incorporate the zero-inflated Poisson (ZIP) model and compare its performance to that of the parameter-free radiation model. All statistical models are developed and executed using the R programming language.

### 3.1 Poisson

Over time, geographers and regional scientists have increasingly favored maximum likelihood estimation (MLE) over ordinary least squares (OLS), particularly in specifying migration as a Poisson-like process. OLS regression, while useful for estimating linear relationships, struggles with the structural characteristics of migration data, particularly due to the preponderance of zero flows. To better address these challenges, contemporary studies have increasingly relied on alternative modeling practices (Cutuli et al. 2024; Huang and Butts 2023).

The shift to Poisson was driven by seminal works such as those by Flowerdew and Aitkin (1982) and Flowerdew and Lovett (1988), as well as by Fotheringham and Williams (1983). The Poisson form of spatial interaction models has grown in popularity, particularly in the analysis of international trade. Silva and Tenreyro (2006) have advocated for Poisson regression, dubbing it the “work horse” of spatial interaction modeling. However, the robustness of Poisson models has been subject to debate, especially when the dependent variable contains a large proportion of zeros. Burger et al. (2009) argued for the utilization of zero-inflated models, as proposed by Lambert (1992), particularly in datasets exhibiting excess zeros.

### 3.2 Zero‑inflated poisson

Zero-Inflated Poisson regression presents a compelling solution to address overdispersion, characterized by the presence of excess zeros in a dataset, by blending two zero-generating processes. Despite its suitability for migration modeling, ZIP regression remains underutilized in the migration literature. However, when examining all possible migration flows between U.S. counties, it becomes evident that a significant proportion, exceeding 90% across various data sources, consists of zero values for a given year (Table 1). This prevalence of zeros underscores the necessity of exploring a zero-inflated regression specification, a notion supported by previous studies.

Bohara and Krieg (1996) and Burger et al. (2009) have laid the groundwork for the application of zero-inflated models in migration analysis. The “zero” component of ZIP models employs a complementary log–log link, diverging from the more conventional logit or probit types. The complementary log–log link function is typically employed when dealing with heavily skewed binary outcomes, where the presence or absence of a particular state or condition is exceptionally rare. This characteristic aptly aligns with the nature of migration data used in our study, further justifying the adoption of ZIP regression as a viable analytical approach.

### 3.3 Radiation

The radiation model, introduced as an alternative approach to modeling human mobility and migration, offers a distinctive conceptualization that portrays mobility as a process of emission and absorption (Simini et al. 2012). Unlike traditional gravity-based migration models, the radiation model operates without the need for parameter estimation, rendering it operationally simpler and more straightforward to implement. This characteristic presents a notable procedural advantage, particularly in scenarios where calibration of model parameters is challenging or impractical. Moreover, the radiation model has demonstrated versatility in capturing mobility dynamics across various temporal and spatial scales, ranging from diurnal variations to national-level movements.

Despite its procedural advantages and versatility, the radiation model’s applicability to migration modeling remains relatively unexplored compared to its applications in short-term mobility studies. Previous investigations predominantly focused on intra-urban commuting patterns and sub-daily movement dynamics, rather than long-term migration processes. Although some studies have conducted systematic comparisons between the gravity and radiation models, these comparisons have primarily centered on short-term mobility rather than migration dynamics (Masucci et al. 2013).

Nevertheless, the theoretical and methodological overlaps between migration and mobility research suggest promising avenues for future exploration and convergence of ideas. Recent scholarship has increasingly recognized the interconnectedness between migration and mobility phenomena (Islam et al. 2021; Piovani et al. 2018), paving the way for potential synergies and cross-fertilization of concepts. As such, future research endeavors could benefit from leveraging insights from both migration and mobility studies to develop comprehensive frameworks that capture the complexities of human mobility across diverse spatial and temporal scales.

### 3.4 Evaluation

Out-of-sample accuracy assessments for each statistical model were conducted using 20 percent of the data for each year and data source combination that was withheld prior to model estimation. We assess the validation results using four metrics: mean absolute percentage error (MAPE), root-mean-square percentage error (RMSPE), mean percentage error (MPE), and mean error (ME) (only used when $M_{ij}$ is equal to 0). The formulas for these measures are shown in Table 2. In these calculations, ${\hat{M}}_{ij}$ and $M_{ij}$ are the predicted and actual migration, respectively, between an origin county i and destination county j.

MAPE measures the average magnitude of errors between predicted and actual values, expressed as a percentage of the actual values. It is useful for understanding the accuracy of predictions in a relative sense, providing an easily interpretable percentage error that can be compared across different datasets or models. However, it can be problematic when actual values are close to zero, leading to high percentage errors.

RMSPE is the square root of the average of the squared percentage errors, giving more weight to larger errors due to the squaring process. This metric highlights models with large errors and is beneficial for identifying when a model performs poorly for certain data points. It provides a more sensitive measure of model accuracy compared to MAPE.

MPE calculates the average of the percentage errors, allowing for the assessment of bias in the predictions by considering the direction of the errors (positive or negative). This metric is useful for understanding whether the model tends to overestimate or underestimate the actual values. A positive MPE indicates a general overestimation, while a negative MPE indicates underestimation, helping diagnose systematic biases in the model.

ME is the average of the errors (differences between predicted and actual values) and is used only when $M_{ij}$ is equal to 0. It helps in understanding the overall bias of the model in absolute terms rather than percentages, showing the average error magnitude. This metric is particularly useful when actual values are zero or very small, where percentage-based metrics may not be applicable.

These metrics collectively provide a comprehensive view of model performance, allowing for detailed evaluation of both the accuracy and bias of predictions across different contexts. Additionally, because the IRS data do not report flows smaller than twenty individuals, an important step in this study will be to validate the migration models for ACS and Census data when 0<M<20.

## 4 Results

**Fig. 3:**
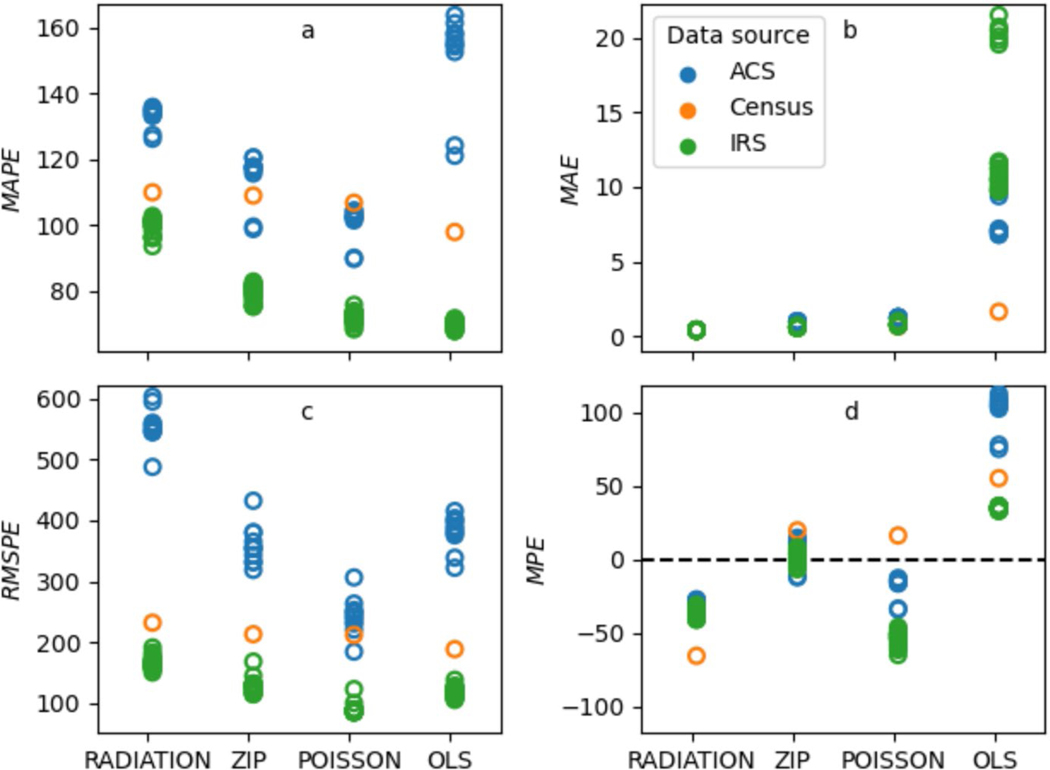
Validation errors by model and data source. Mean absolute error (MAE; panel b) is only calculated when observed migration values are equal to zero (M = 0). All other metrics are calculated when migration values are greater than zero (M > 0)

The four error metrics for the four migration models are summarized in Fig. 3. Overall, the IRS datasets exhibit lower error rates compared to the Census and ACS datasets across most metrics, except for the mean absolute error (MAE) for the ordinary least squares (OLS) model (Fig. 3b). The Census models generally demonstrate higher accuracy than the ACS datasets, although they show more bias when considering the mean percentage error (MPE) metric. In comparing the models, the OLS model appears robust based on the mean absolute percentage error (MAPE) measurement. However, the larger absolute errors, coupled with higher root-mean square percentage error (RMSPE) and MPE values, suggest it is less desirable overall. The radiation model excels in the MAE metric but performs poorly on other metrics. The Poisson model demonstrates strong performance in the MAPE and RMSPE metrics, even outperforming the zero-inflated version in some cases. Nonetheless, the zero-inflated Poisson model exhibits lower bias and nearly comparable values across the other error metrics, supporting its use for migration modeling.

**Fig. 4:**
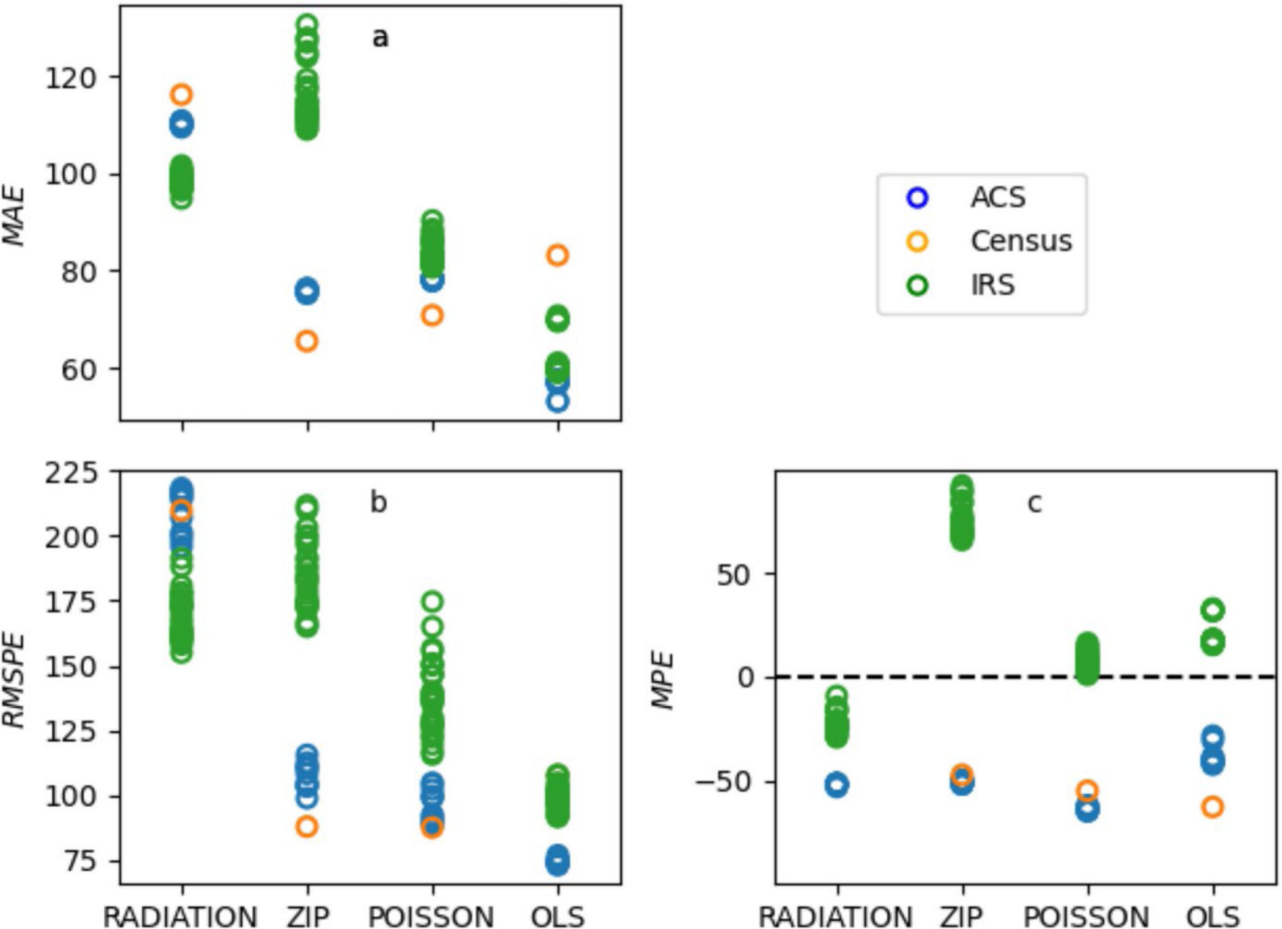
Validation errors for migration values > 20 only. After excluding small migration flows from ACS and Census, IRS models generally perform worse using the MAPE and RMSPE metrics. MAE is undefined when migration flows are > 0

Figure 4 presents model accuracy for migration flows greater than 20 people, providing a fair comparison between models derived from IRS data—which excludes those small migration flows by default—and ACS and Census, which do not. Overall, model performance is improved for ACS and Census models, showing lower MAE and RMSPE values compared to Fig. 3. The relative advantage of IRS data presented in Fig. 3 is reversed for the ZIP and POISSON models and reduced for the OLS models but persists for the radiation models (Fig. 4a and b). That is, IRS models perform worse than ACS and Census models in several cases after truncating small values. Interestingly, IRS models do generally show less bias than ACS and Census for the truncated data, with the ZIP model being the exception (Fig. 4c). MPE values are similar for the radiation model with and without truncation, indicative of a model form with consistent bias across small and large values. The other three models show very different patterns. ACS and Census models possess a clear negative bias (i.e., underpredict) for values > 20, while in most cases the bias was positive or only slightly negative with the full datasets (Fig. 3d). The OLS performs markedly better when small migration values are removed, however the bias of the models shift from positive to negative for ACS and Census. The comparison of model performance with and without truncation highlights challenges when comparing results across studies that use different data sources.

**Fig. 5:**
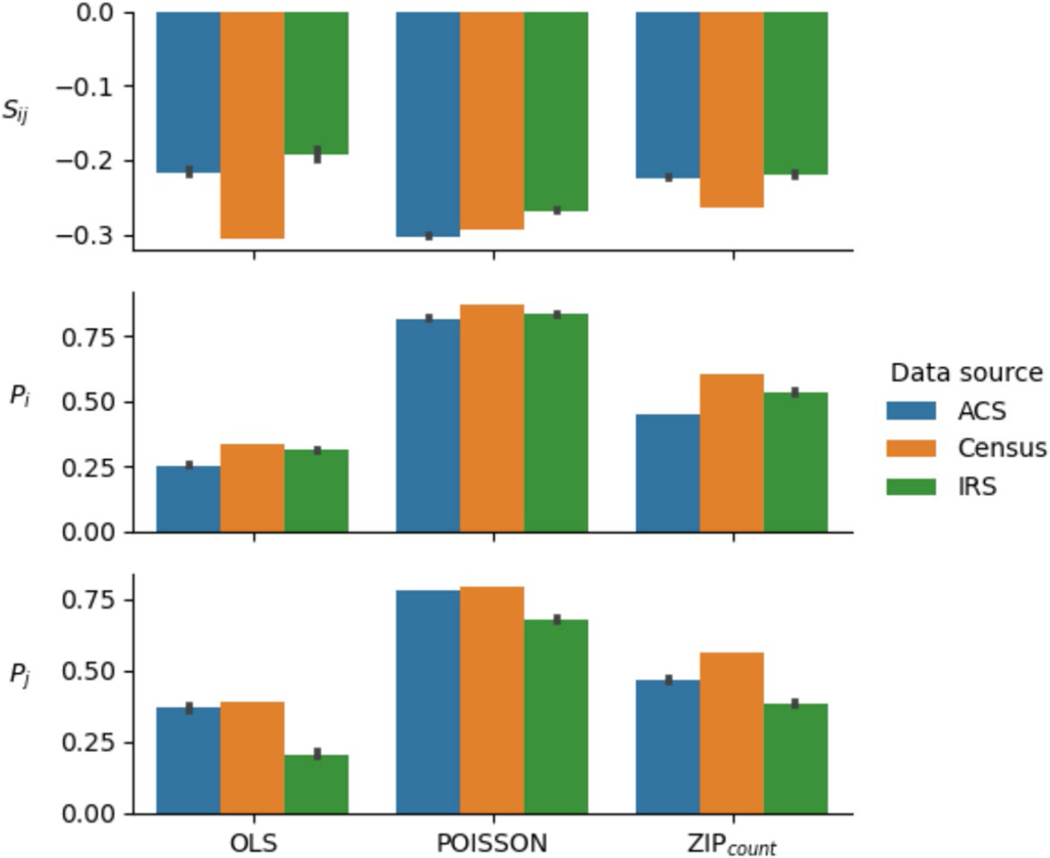
Variable coefficients by data source. Bars show the average of all years; error bars show the range

In Fig. 5, we compare the model coefficients for the OLS, Poisson, and zero-inflated Poisson (ZIP) models across the three primary coefficients considered in the model for each of the three datasets. Although the differences in these coefficients are statistically significant, the functional differences are generally quite limited. In each case, the coefficients consistently point in the same direction and are often within 5% of one another. This suggests that, while the models may appear to perform similarly based on their coefficients, the substantial variances in error metrics beneath the models indicate otherwise.

The similarities in coefficients across different models and datasets, despite these error variances, highlight an important issue in migration modeling. Models with similar coefficients can produce geographically divergent predictions due to underlying discrepancies in error distribution and data quality. This underscores the necessity for careful model validation and selection based on comprehensive error analysis rather than solely on coefficient similarity. Understanding these nuances is crucial for researchers and policymakers who rely on accurate migration models to inform decisions and develop strategies. The geographical divergence in predictions, despite similar coefficients, can have significant implications for regional planning and resource allocation, emphasizing the need for a nuanced approach to model interpretation and application.

## 5 Conclusion

This study provides a comprehensive analysis of various migration models using three distinct datasets examining how different model specifications respond to the unique characteristics of each data source. The analysis focused on comparing the performance of ordinary least squares (OLS), Poisson, zero-inflated Poisson (ZIP), and radiation models across multiple error metrics. Overall, the IRS datasets, despite lacking demographic details, exhibited lower error rates compared to the Census and ACS datasets across most metrics, except for the MAE for the OLS model, particularly in handling total migration flows. The Census models generally demonstrated higher accuracy than the ACS datasets, although they showed more bias when considering the MPE metric. In comparing the models, the OLS model appeared robust based on the MAPE measurement. These findings do not imply overall superiority, as the suitability of each dataset depends on the research question: IRS data are well-suited for estimating total migration volumes, while ACS and Census data provide richer demographic insights.

Model comparisons reinforce the importance of choosing appropriate specifications for migration modeling. While OLS appeared robust based on the MAPE measurement, its high absolute errors and sensitivity to zero flows make it a weaker option for long-range projections where small errors can accumulate over numerous time steps. The inability of OLS to incorporate zero flows effectively due to the undefined nature of the natural logarithm of zero is a fundamental limitation given that more than 90% of all possible county-to-county migration flows are zero each year. The radiation model performed well on MAE but exhibited weaknesses in other metrics, limiting its broader applicability. The Poisson model demonstrated strong predictive accuracy, though the zero-inflated Poisson model better accounted for the prevalence of zero migration flows, supporting its use in migration studies where excess zeros are a concern. The usage of negative binomial and zero-inflated negative binomial models when there is overdispersion (de Silva and de Sousa 2023) is likely also an effective modeling technique while maintaining interpretability and computational efficiency.

Our findings underscore the trade-offs between dataset completeness and demographic richness. The IRS datasets, while more comprehensive in capturing total migration flows, lack demographic details, making them less suitable for analyses focused on population characteristics. However, their completeness makes them well-suited for models emphasizing broad migration patterns. In contrast, Census and ACS data provide a more nuanced understanding of migration dynamics among different population groups, making them indispensable for studies on race, ethnicity, and socioeconomic disparities. However, the Census dataset lacks continuity, while ACS data, despite providing rolling averages, can introduce biases due to sampling variability.

Given these distinctions, using multiple datasets with varying characteristics is essential for a comprehensive understanding of migration dynamics. Each dataset offers unique insights that complement one another. The IRS dataset provides critical economic perspectives on migration through changes in household addresses and aggregate adjusted gross income. Census data offer detailed demographic breakdowns, allowing for an in-depth analysis of migration patterns across racial, ethnic, and socioeconomic lines. The ACS, with its continuous collection and rolling averages, captures more recent migration patterns, enabling researchers to identify shifts and trends over time. The choice of dataset should therefore be guided by the specific research question and modeling approach, rather than an assumption that one dataset is inherently superior to another.

Our findings also contribute to the ongoing reassessment of the radiation model’s role in migration research. Although the model provides an appealing alternative to gravity-based frameworks by framing migration as a process of emission and absorption, its empirical performance in our analysis is mixed. Specifically, while the radiation model excels at predicting zero migration flows, it performs poorly when estimating flows above the suppression threshold (M ≥ 20), diverging from claims about its general applicability for migration forecasting. This performance gap, combined with its theoretical simplicity and minimal data requirements, suggests that the radiation model may still have value in contexts with sparse data or limited computational capacity. However, for researchers aiming to model detailed or demographically nuanced migration patterns, more advanced alternatives should be considered. Emerging approaches such as machine learning (Bircan and Salah 2024), Bayesian hierarchical models (Cutuli et al. 2024), temporal dynamics (Qi and Bircan 2023; Beyer et al. 2022), and geographically weighted techniques (Yu 2024) show promise in improving predictive accuracy.

Recent work using passively collected “digital-trace” data such as Facebook Advertising mobility panels, mobile-phone location histories, and Google Location Services has opened alternative avenues for estimating domestic migration flows at fine temporal scales (Alexander et al. 2019). While these sources expand coverage and timeliness, they rely on convenience samples and require post-stratification, so researchers should treat them as complements rather than substitutes for IRS, ACS, or Census flow tables. Our analysis highlights that both data source and model selection exert measurable influence on migration flow estimates, particularly when modeling at the county level in the U.S. context. Researchers must be attentive not only to the statistical performance of models but also to the assumptions embedded within each dataset, such as flow suppression thresholds and population coverage. The IRS dataset offers robust performance for aggregate flows but lacks demographic resolution; the ACS and Census data provide richer individual-level detail but introduce variability through small-flow estimates. Future work should continue to explore model-data fit systematically, ideally through modular frameworks that allow researchers to adapt model complexity to data constraints. Such an approach would support more transparent, purpose-driven modeling, critical for demographic forecasting, regional planning, and policy evaluation.

## Figures and Tables

**Table 1:** County-to-county migration datasets used in this study

Data source	Time period	# of unique datasets	Migration interval	Suppressions	Percent of flows that are 0

ACS	2005 to 2020	11	Annual (five-year average)	Small flows*	97.3
Census	2000**	1	Five years	Unknown***	92.9
IRS	1990 to 2020	30	Annual	Flows < 20	99.2

* Flows containing only one or two people from different households, only one or two people in group quarters, or one person in group quarters and the rest from a single household are suppressed

** The 2000 migration data are the only county-to-county vintage still available on the Census web page. Earlier decennial migration data are available through the National Historical Geographic Information System (Manson et al., 2024)

*** Technical documentation of the long-form Census questionnaire is available but makes no specific mention of migration data suppression procedures

**Table 2:** Formulas for evaluation metrics

$\;\text{MAPE}\;=\frac{\sum \frac{\left|{\hat{M}}_{ij}-M_{ij}\right|}{M_{ij}}}{n}\times 100$ Mean absolute percentage error (MAPE)	$\;\text{RMSPE}\;=\sqrt{\frac{\sum {\left(\frac{{\hat{m}}_{ij}-M_{ij}}{M_{ij}}\right)}^{2}\times 100}{n}}$ Root-mean-square percentage error (RMSPE)

$\;\text{MPE}\;=\frac{\sum \frac{{\hat{m}}_{ij}-M_{ij}}{M_{ij}}}{n}$ Mean percentage error (MPE)	$\text{ME}=\frac{\sum {\hat{M}}_{ij}-M_{ij}}{n}$ Mean error (ME), calculated only when $M_{i}j=0$
